# Equity Promoting Integrated Care: Definition and Future Development

**DOI:** 10.5334/ijic.7614

**Published:** 2023-10-19

**Authors:** Paul Wankah, Dara Gordon, Simone Shahid, Shivani Chandra, Ibukun-Oluwa Abejirinde, Rosanra Yoon, Walter P. Wodchis, Patricia O’Campo, Carolyn Steele Gray, Nancy Clark, James Shaw

**Affiliations:** 1 University of Toronto, CA; 2 Women’s College Hospital, CA; 3 Toronto Metropolitan University, CA; 4 Institute for Better Health, Trillium Health Partners, CA; 5 Unity Health Toronto, TO; 6 Lunenfeld-Tanenbaum Research Institute, Sinai Health, CA; 7 University of Victoria, CA; 8 University of Toronto, Women’s College Hospital, CA

**Keywords:** Integrated care, Health equity, Health system transformation, Implementation science, Social justice

## Abstract

Over the last three decades, integrated care has emerged as an important health system strategy to improve population health while addressing the unique needs of structurally marginalised communities. However, less attention has been given to the role of integrated care in addressing issues related to inequities in health and health care. In this commentary we introduce the concept of Equity Promoting Integrated Care (EPIC) that situates integrated care in a social justice context to frame the actions necessary to center equity as a priority for integrated care. We suggest that efforts to advance the design and implementation of integrated care should focus on three avenues for future research and practice, namely, the collaborative mobilization of a global network of integrated care stakeholders to advocate for social justice and health equity, investing in equity-focused approaches to implementation science that highlight the importance of social concepts such as colonialism and intersectionality to advance the theory and practice of implementing EPIC models of care, and leveraging innovative approaches to measuring equity-related aspects of integrated care to inform continuous improvement of health systems.

Integrated care has been a focus for health systems strategy and policy around the world for the past three decades [1,2,3], generating a large body of research on its role in health care transformation and sustainability [4,5]. Integrated care is an approach that promotes collaboration across organizational and professional boundaries to provide more connected care for patients, family and caregivers in their local communities. Integrated care specifically aims to redress system fragmentation and inefficiencies which often create health care inequities. Systematic reviews suggest that many models of integrated care show clear benefit in proximal outcomes such as enhanced access to care and patient satisfaction [5,6,7], along with some evidence documenting cost savings and reduced health system usage for certain patient groups [8]. However, less attention has been given to the role of integrated care in addressing issues related to inequities in health and health care, defined as systematic, unjust, and avoidable differences in health for population groups who experience structural marginalization [9].

Equity in access to and outcomes of health care represents a central goal of health care systems, supporting efforts to achieve improvements in population health for all communities served [10]. However, health care systems face considerable challenges to achieving equity in access and outcomes due to recurring societal factors such as out-of-pocket expenses that impact affordability of care, and increasing population diversity with emerging disparities. These structural conditions have been heightened in the context of COVID-19 and primary healthcare crisis, where the health workforce may be less motivated to uptake equity-oriented changes in their routine practices [11]. In this commentary we introduce the concept of Equity Promoting Integrated Care (EPIC) that situates integrated care in a social justice context to frame the actions necessary to center equity as a priority for integrated care. An operational definition for EPIC is needed to understand its importance and to show how it can be mobilized to redress inequities in health and health care.

## Defining integrated care

Integrated Care has been defined in many different ways that converge on a common set of shared principles and practices for care [2,12]. In the broadest sense, integrated care includes changes that enhance teamwork and patient centeredness across the dimensions of policy, health systems, organizations, and health care provider practices [4,13,14]. In this commentary we align with a recent definition of integrated care from Shaw et al (2022), who defined integrated care as “a collection of individuals and groups (including patients, caregivers, health care providers, managers and other actors), and their organizations, working together to provide equitable and culturally safe care for patients that is coordinated as best as possible along informational, relational and care management dimensions” [15]. This definition emphasizes the importance of local context and the ongoing efforts to refine models of integrated care at local levels. Despite the value of this definition in emphasizing the inclusion of patient and family/caregiver voices, as well as the importance of working across sectors to address the needs of communities which cannot be addressed in isolation, this and other existing definitions do not adequately address inequities in health and health care. We propose to build upon this definition in the effort to provide a conceptual basis for Equity-Promoting Integrated Care.

## Defining Equity Promoting Integrated Care

Margaret Whitehead (1991) defined health equity as the state in which everyone has a fair opportunity to attain their full health potential [9]. There has been growing calls for governments and health care systems to address health inequities [16,17,18]. The effort to promote health equity through the implementation of integrated models of care begins with a clear understanding of the ways in which social, political and historical systems generate health inequities in the first place [19,20]. A clear understanding of these root causes provides a crucial input to the effort to design models of care with higher potential to intervene in the pathways through which health inequities are produced [21].

Scholarship on the fundamental causes of health inequities has made important advances, clarifying areas of focus for policy, advocacy, and practice in health systems [22,23]. Specifically, scholarship has highlighted the intersecting systems of oppression that are upheld by social institutions and policies (including those in health care) that have clear detrimental effects on health. For example, research has documented the exclusionary and damaging effects on health of poverty [24], sexism [25], racism [26], hetero-sexism [27], ableism [28], and white supremacy [29,30], with their intersecting influence causing lasting damage to the health of populations [31]. The recently described Health Power Resources Theory (HPRT) describes the central role of power in determining who has access to which resources, and the structurally determined capacities of individuals and communities to mobilize resources toward achieving better health [32]. Where systems of oppression actively exclude communities from health care and opportunities to promote health, communities are then deprived of the resources necessary to achieve improved health outcomes.

In summary, health inequities are generated by social structures that uphold intersecting systems of oppression that privilege some communities and actively marginalize other communities through individual, interpersonal, and systemic pathways of oppression and discrimination. These pathways of oppression and discrimination limit the power and resources available to marginalized communities and reduce their capacity to achieve their full health potential.

In the context of social structures upholding systems of oppression that determine health inequities, we define Equity Promoting Integrated Care (EPIC) as referring to models of care that (a) are explicitly focused on enhancing the health status of members of communities adversely affected by intersecting systems of oppression, (b) aim to re-balance power by including and platforming the voices of community members in governance and organizational decision-making, (c) enhance the capacity and resources of community members to more effectively engage in self-defined healthy living, (d) coordinate access to health, social, and other services across sectors, (e) deliver culturally safe and trauma and violence-informed health services, and (f) advocate for structural changes to intersecting systems of oppression that produce privilege and marginalization among local communities.

## Developing Equity Promoting Integrated Care

The concept of EPIC models of care offers a novel focus for efforts to advance the design and implementation of integrated care, and as such demands novel conceptual and practical efforts to plan and study their implementation. We suggest three avenues for future research and practice on this topic, which we outline here.

First, we suggest a collaborative mobilization of the extensive global network of integrated care researchers, policymakers, providers, patients, caregivers, and other community members to advocate for social justice and health equity. As one important direction for advocacy, the global network of integrated care stakeholders needs to work toward changes to the social structures that uphold intersecting systems of oppression in policy and other social institutions. The formal and informal rules that govern which patients experience easy access to health care, who holds powerful jobs in health systems, and who gets to shape the strategic directions of health care need to be disrupted if the changes we outline here are to be achieved.

As a second important direction for advocacy, the global network of stakeholders can advocate for explicit attention to disrupting systems of oppression and commitment to social justice in the implementation and improvement of integrated models of care. These will help to shape a more supportive context for EPIC models of care.

As a final important direction for advocacy, current global movements towards patient-centered care mean greater involvement of various actors such as the public, patients, family and the community in research as well as the organization and delivery of care [33]. Inclusion of peer-led programs as well as care coordinators may address some structural barriers to integrated care. However, limited inclusion of structurally marginalized patients, families, and caregivers in the organization and delivery of care may perpetuate inequities between groups [34]. Hence, it is critical to develop and promote inclusive strategies that leverage patient and family groups with living experiences of structural marginalization to dismantle inequities related to current health care contexts.

Second, we suggest that integrated care community members invest in equity-focused approaches to implementation science for EPIC models of care. Although theories, models and frameworks of implementation science that focus on health equity are emerging [4,35,36,37], little attention has been paid to social theory that articulates the implications of concepts such as colonialism and intersectionality for the theory and practice of implementing EPIC models of care [38]. Building the literature on equity-focused implementation science specifically for EPIC models of care will bring distinct lines of implementation research together in service of social justice and health equity.

Third and finally, we suggest that integrated care researchers invest in novel approaches to measuring the equity-related effects of integrated models of care. It is well understood that health systems must measure specific health inequities if they are to monitor progress in addressing those inequities [39]. However, health inequities have not been a common metric of progress in the area of integrated care [40,41]. In a 2017 systematic review, Sunderji et al [42] synthesized 148 unique measures of integrated care with only three equity-focused measures identified, namely, (a) care provision responds to disadvantaged client populations, (b) client barriers in accessing care, and (c) provider attitudes to mental health. Building on this past work, we suggest that further work is needed to better understand measurement strategies, measurement frameworks and indicators of equity in the field of integrated care.

## Conclusion

All over the globe, integrated care systems are increasingly supporting the improvement of community and population health. We contend that specific attention to health inequities is necessary to adequately address social justice and promote equity in access to and outcomes of integrated care. A clear definition of EPIC provides a basis for shared understanding of this concept and a foundation for advocacy, research, policy, and practice to advance equity centered integrated care for all.
